# A qualitative study on healthcare professionals’ perceived barriers to insulin initiation in a multi-ethnic population

**DOI:** 10.1186/1471-2296-13-28

**Published:** 2012-07-04

**Authors:** Yew Kong Lee, Ping Yein Lee, Chirk Jenn Ng

**Affiliations:** 1 Department of Primary Care Medicine, Faculty of Medicine, University of Malaya, 50603, Kuala Lumpur, Malaysia; 2 Department of Family Medicine, Faculty of Medicine and Health Sciences, Universiti Putra Malaysia, 43400 UPM, Serdang, Selangor, Malaysia

## Abstract

**Background:**

Nationwide surveys have shown that the prevalence of diabetes rates in Malaysia have almost doubled in the past ten years; yet diabetes control remains poor and insulin therapy is underutilized. This study aimed to explore healthcare professionals’ views on barriers to starting insulin therapy in people with type 2 diabetes.

**Methods:**

Healthcare professionals consisting of general practitioners (n = 11), family medicine specialists (n = 10), medical officers (n = 8), government policy makers (n = 4), diabetes educators (n = 3) and endocrinologists (n = 2) were interviewed. A semi-structured topic guide was used to guide the interviews by trained facilitators. The interviews were transcribed verbatim and analysed using a thematic analysis approach.

**Results:**

Insulin initiation was found to be affected by patient, healthcare professional and system factors. Patients’ barriers include culture-specific barriers such as the religious purity of insulin, preferred use of complementary medication and perceived lethality of insulin therapy. Healthcare professionals’ barriers include negative attitudes towards insulin therapy and the ‘legacy effect’ of old insulin guidelines; whilst system barriers highlight the lack of resources, language and communication challenges.

**Conclusions:**

Tackling the issue of insulin initiation should not only happen during clinical consultations. It requires health education to emphasise the progressive nature of diabetes and the eventuality of insulin therapy at early stage of the illness. Healthcare professionals should be trained how to initiate insulin and communicate effectively with patients from various cultural and religious backgrounds.

## Background

The incidence of diabetes is increasing globally [1,2] particularly in the Asia Pacific region [3]. Currently, Malaysia has the highest prevalence rate of diabetes (11.6%) in the Western Pacific region [4] and it is the 10^th^ highest in the world [2]. This alarming rise in the prevalence of diabetes is has been attributed to increasing affluence, rapid urbanization, and a diet rich in carbohydrates [4]. In addition, Malaysia having an upper-middle-income economy, high treatment costs of diabetes and its associated complications have imposed a substantial healthcare burden to her already stretched health system [5]. As such, in 2010, the Ministry of Health of Malaysia has included diabetes as a priority area in the National Strategic Planning for Non-Communicable Diseases [6].

Malaysia has a dual-sector healthcare system comprising government-subsidised public healthcare facilities and more expensive, private healthcare clinics and hospitals [7]. Patients are free to choose where they receive treatment, but patients prefer to seek treatment in government facilities as treatment costs are lower there compared to private clinics. Out-of-pocket expenditure was 40.5% of total healthcare expenditure in 2009 [8]. In primary care, the private sector comprises mostly solo general practice clinics [9] whilst public primary care consists of government health clinics and university-based primary care clinics. There are about five times more private primary care clinics compared to the public sector in Malaysia [10]. Primary care practice is expected to play a gatekeeper role for secondary care referrals [10].

The quality of diabetes care in private primary care clinics is doctor-dependent as clinics are mostly solo practices employing nursing aides with little formal training [9,10]. In the public sector, the quality of care also varies, often better in the urban health clinics and university-based primary care clinics due to the presence of family medicine specialists and multidisciplinary diabetes teams [9]. Clinics in the public sector provide more comprehensive diabetes services but have a high patient load compared to private clinics [10].

The majority of patients with diabetes are managed in the government facilities; the rest are treated by private general practitioners or take complementary and alternative medications [4]. Despite the established risk of microvascular complications associated with hyperglycaemia [11], diabetes control remains poor in the Malaysian primary care setting. Eighty percent (80%) of the patients in the private [12], and 69.1% in the public setting, failed to achieve an HbA1c level of less than 7.0% [13]. One main reason for poor control is the lack of timely treatment intensification such as initiation of insulin therapy [14]. In a community based national health survey, only 7.2% of Malaysian patients with type 2 diabetes used insulin, either alone or as combination therapy [4], compared to 36% in the United States [15].

The Malaysian clinical practice guideline (CPG) for type 2 diabetes was last updated in 2009 and insulin therapy was stated as part of the treatment algorithm [16]. However, there was no mention of how insulin initiation could be implemented in the local healthcare setting. Recognising this gap, a practical guide for insulin therapy was developed in 2010 and a section was dedicated specifically to addressing patients’ barriers to insulin initiation [17]. However, the recommendations are based on Western data and experts’ opinions. There is little research on what barriers the multi-ethnic Malaysian patients with type 2 diabetes face when deciding to initiate insulin. Studies from other countries have described various barriers to insulin initiation such as needle phobia, low self-efficacy and feelings of personal failure [18,19]. It is reported that up to a third of patients are unwilling to start insulin therapy when advised to do so [18,19].

Insulin can only be prescribed by doctors in Malaysia and can be initiated at either primary or secondary care settings. Nurse educators play an important role in the public sector as doctors would refer patients to the nurses for education and instruction after prescribing insulin. On the other hand, private doctors often seek help from diabetes educators, who are sponsored by pharmaceutical companies or non-governmental organizations.

Malaysia’s multi-cultural society consists of three main ethnic races (Malays, Chinese and Indians) and many other smaller ethnic groups [20], which may influence how both healthcare professionals and patients view insulin therapy. This study, therefore, aimed to identify barriers to insulin initiation from the healthcare professionals’ perspective. It is only through understanding the barriers to insulin initiation that healthcare professionals can address patients’ concerns and help them make decisions about starting insulin. This study was part of a larger three-year complex intervention study which aimed to develop a local patient decision aid for insulin initiation.

## Methods

### Design

Qualitative, semi-structured interviews and focus groups were used to identify and explore barriers to insulin initiation as viewed by healthcare professionals. A qualitative methodology was used as it allowed us to explore and probe the beliefs, experiences and views of the healthcare professionals concerning insulin initiation as encountered in their respective local practices [21].

For the focus group discussions, we selected and grouped the participants based on their practice background and location to ensure homogeneity and to capitalise on their shared experiences [22]. The focus groups consisted of two groups of private primary care doctors (n = 4, n = 7), public family medicine specialists (n = 8) and public medical officers in a university hospital primary care clinic (n = 8). In-depth interviews were used for key opinion leaders, such as government policy makers, and also for those who were unable to commit to a focus group session due to their busy schedule. The use of in-depth interviews, focus group discussions and field notes provided the basis for the triangulation of the data. Although all interviews were conducted in English, some participants used Malay-language words and phrases during the interviews as Malay is the national language.

### Setting

The study was conducted amongst healthcare professionals who provided diabetes care in the three healthcare settings in Malaysia: the government health clinics; government university-based primary care clinic and hospital; and private general practice (GP) clinics and hospitals. Key government policy makers who were involved in shaping the national diabetes strategic plans were also interviewed. A spectrum of practice experience was represented. The healthcare professionals came from three different states and from both urban and semi-rural locations.

### Participants, recruitment, sampling

Purposive sampling was used whereby we identified stakeholders who were involved in insulin initiation in both primary and secondary care and contacted healthcare professionals from each stakeholder group. They included: endocrinologists, family medicine specialists, government policy makers, general practitioners, government medical officers and diabetes nurse educators. A pattern of snowball sampling developed as the participants named individuals and organizations who were involved in diabetes care particularly healthcare professionals who initiated insulin therapy. Sample size was determined by data saturation whereby interviews were stopped when no new themes emerged from the interviews.

### Data collection

An interview topic guide was developed based on literature review, clinical knowledge and research experience (Table 1). The same guide was used for both individual and focus group discussions. Participants consented to be audio-recorded and interviews were carried out by either one of two researchers who were trained to conduct qualitative interviews and facilitate focus groups. Care was taken to avoid potential participant response bias by avoiding, whenever possible, having participants interviewed by close acquaintances, lecturers or colleagues. An assistant took detailed notes and observed non-verbal cues during the interviews and these observations acted as field notes. Between October 2010 and May 2011, we conducted ten 30–40 min individual interviews and four one hour-long focus groups. We stopped data collection when data saturation was reached for both interviews and focus groups. The interviews and focus groups were audio-recorded and transcribed verbatim. The transcripts were checked for accuracy and used as data for analysis.

**Table 1:** Barriers to insulin initiation interview/focus group topic guide

·→	Is starting insulin a difficult decision for your patients?
·→	How do they feel when making this decision?
·→	What are the things that patients consider before they decide whether or not to start insulin?
	Information
	Values
	Influence from others
·→	What barriers do you face when advising them to start insulin?
·→	What kind of help do you need to overcome these barriers?
·→	What barriers do you face when shaping policies on insulin treatment? (additional question for policy makers)

### Data analysis

A hermeneutic-phenomenological approach was employed when analysing the data, which was viewed as being both descriptive and interpretive [23]. The interpretive focus of hermeneutics occurred from the ‘outside’, whereby two of the researchers’ backgrounds as clinicians influenced how they not only interpreted the data but also how their interactions with the participants during interviews were influential in constructing the text. The hermeneutic perspective was also acknowledged on the ‘inside’, from the perspective of the participants, whereby data was viewed as consisting of how participants interpreted barriers to insulin initiation, both from their perspective as healthcare providers, and also on behalf of their patients [24]. The researchers familiarised themselves with the data by reading and re-reading the transcripts. Three researchers coded two transcripts (interviews with a primary care physician and a government policy maker) independently and created a list of nodes (themes). Subsequently, the researchers used this framework to code (label) the two other transcripts individually. The coding was then compared for inter-rater consistency and any coding discrepancies were resolved by discussion until consensus was reached on the list of nodes and the coding descriptions. The finalised list of nodes and coded transcripts were imported into Nvivo9 software which formed the basis for future coding.

The remaining transcripts were distributed among the three researchers (YKL, PYL, CJN) and coded individually. Any new nodes emerging during coding were added to the list upon consultation with the other researchers. The list of nodes was regrouped into larger categories as a pattern of themes emerged from the data.

Two of the researchers (CJN, PYL) are family medicine specialists and the third is a postgraduate psychologist (YKL) and thus data analysis was from both clinical and non-clinical perspectives. The researchers constantly reflected and debated on the potential biases which they might carry with them due to their backgrounds to improve credibility of the analysis.

### Ethics approval

This study received ethics approval from the Medical Research and Ethics Committee, Ministry of Health, Malaysia.

## Results

A total of 38 healthcare professionals participated in the study. Besides individual interviews, two focus group discussions were conducted with general practitioners in private practice (n = 7; n = 4), one focus group with family medicine specialists from public health clinics (n = 9), and another focus group (n = 8) with medical officers from a public hospital-based primary care clinic. Participants’ demographic data are shown in Table 2.

**Table 2:** Demographic profile of participants

**Characteristics**	**Number (n = 38)**	**%**	**Mean ± SD (Range)**
Age			47.0 ± 9.9 years (30–66 years)
Sex			
Female	29	76.3	
Male	9	23.7	
Ethnicity			
Malays	13	34.2	
Chinese	12	31.6	
Indians	10	26.3	
Others	3	7.9	
Professional background			
General practitioner	11	36.7	
Family medicine specialist	10	33.3	
Government policy maker	4	13.3	
Diabetes nurse educators	3	10.0	
Endocrinologists	2	6.7	
Healthcare sector			
Public	24	63.2	
Private	14	36.8	

Three main categories of barriers emerged from the analysis and are reported below: patient barriers, healthcare professional barriers and system barriers (Table 3). Quotations are verbatim whereby colloquialisms and Malay-language words (with translations), if any, are not re-worded in order to give perspective on Malaysia’s multi-lingual setting.

**Table 3:** Barriers to insulin initiation faced by Malaysian healthcare professionals

**Patient barriers**	
·→	Fear of side effects and pain
·→	Misconceptions about insulin
	Insulin is lethal
	Insulin is a punishment
	Insulin is a stigma
	Insulin is a medication for old people
	Insulin causes sexual dysfunction
	Insulin is unlawful for Muslims
·→	Seeking alternative treatment
·→	Lack of knowledge and self efficacy
·→	Negative influence from family members
**Healthcare professional barriers**	
·→	Negative attitudes towards insulin
·→	Lack of motivation and confidence
·→	Training-related barriers
·→	Conflicting advice from the healthcare professionals
**System barriers**	
·→	Lack of continuity of care
·→	Lack of manpower
·→	Lack of resources
·→	Language barriers

### Patient barriers

The participants highlighted a range of barriers faced by patients when starting insulin. The list includes fears associated with insulin; patients’ perceptions of insulin; lack of knowledge and self-efficacy.

#### Fear of side effects and pain

The healthcare professionals found that patients’ fear of side effects, such as hypoglycaemia and weight gain, were common barriers faced by patients.

"“(Patients are) afraid of hypo. Because they have seen people with hypo, it’s so bad. They lost consciousness and they talk nonsense and all that.”"

"Family medicine specialist, public health centre"

"“The youngsters especially…they are very worried about weight gain.”"

"Medical officer, public university primary care clinic"

Other emotional factors that influenced patients’ decision on starting insulin included patients’ fear of needles and pain.

"“All of us are brought up (to believe that) injection is pain. So a lot of them have a (pause) idea that it is associated with pain”"

"Diabetes nurse educator, public university hospital"

"“The moment we say injection, for them, injection is the long needle…the big needle. So that’s the idea…”"

"Medical officer, public university primary care clinic"

#### Misconceptions about insulin

##### Insulin is lethal

The healthcare professionals cited that patients’ perceived insulin as a drug with ‘lethal’ complications. Patients believed that they would die soon after initiating insulin because they observed that the disease deteriorated in other patients soon after insulin initiation. As a result, they perceive that insulin is the cause of severe diabetic complications.

"“It’s..it’s a…especially among the elderly patients, they'll be told that when they reach the stage where they need insulin, err…that's one foot in the grave already”"

"Endocrinologist, private hospital"

"“…so they (patients) feel that the moment they put insulin, after a few years is kidney damage, then dialysis. So they have that fear, every time they’ll ask us, “Doctor if I use insulin, will my kidneys get damaged?””"

"General practitioner, private practice"

##### Insulin is a punishment

Some patients perceived insulin as a ‘punishment’ to them. The healthcare professionals believed that this could be due to doctors framing insulin as a penalty for failing to control their disease. A doctor quoted a patient as saying *“…one doctor very* garang *(‘fierce’), you know, scolded me because my sugar is like this. And said if it’s not okay, I’ll start you on insulin. So for (me) it’s a punishment”.*

##### Insulin is a stigma

According to the healthcare professionals, using insulin might be perceived by some patients as a stigma as they associated needles with drug abuse. One endocrinologist observed that,

"“I’ve no idea why they think they will be addicted to insulin…I don’t know what it is about insulin perhaps it’s the fact there’s a needle and I don’t know whether they think it’s dadah (drugs) or what, but very often like, ‘Oh does it mean sampai mati saya kena ambil (I have to take insulin until I die) or umm… does it mean I can’t come off it, imply that I addicted…dependent on it…’”."

Patients also worried about having to inject insulin during social functions where they would be surrounded by other people.

"“How to inject in front of public, like I go for dinner, I’m going to attend a dinner with everybody on the round table. So when can I inject myself…am I going to inject myself in public…or where can I go myself injection?”"

"Medical officer, public university primary care clinic"

##### Insulin is a medication for old people

Younger patients viewed diabetes as an ‘old people’s disease’ and considered insulin as only needed for the elderly.

"“They (young people) got a stigma… Because you see, insulin, and diabetes, is old peoples’ disease.”"

"General practitioner, private practice"

##### Insulin causes sexual dysfunction

Insulin was also associated with men’s sexual dysfunction.

"“They think by taking this tablet (diabetes medication), it makes them, you know… ED (erectile dysfunction), so no injection, any medicine, or any injection”"

"General practitioner, private practice"

##### Insulin is unlawful for Muslims

Muslim patients were concerned over the origin of insulin as many still believed that it was a porcine derivative, which is unlawful under Islamic religious law.

"***“***I think they were thinking that the insulin is from, what do you call this, non-halal (‘lawful’)…ah…products”"

"Family medicine specialist, public health centre"

#### Inconvenience in starting insulin

Patients also perceived insulin therapy as inconvenient and interfering with their lifestyle.

"“Yeah, I think some of them said inconvenience because they said uh especially those already retired, they actually want to go into you know, different-different places, different child each month, or go to the relatives’ house and all that. So, yeah to bring, they thought that they actually have to keep that in the fridge all the time. So, it’s actually inconvenient for them. Also for injection lah. I mean, if they’re actually go out, injections probably a problems for them.”"

"Family medicine specialist, public health centre"

#### Seeking alternative treatment

Complementary and traditional medications for diabetes were also preferred for diabetes control.

"“And when you tell them, your diabetes has come to a stage where you need, er, injections, they will say they have uh…these herbs and so on. They want to try out herbs first.”"

"General practitioner, private practice"

#### Lack of knowledge and self efficacy

Patients with diabetes often considered starting insulin therapy as a complex task and this caused patients to delay insulin therapy. Patients felt overwhelmed by the instructions and were not confident to handle injections.

"“…let’s talk about older people, for the older people, they always know insulin is more complicated rather than just following medicine. So they always say that I cannot handle it, so I don’t want it.”"

"Diabetes nurse educator, private practice"

Patients’ lack of self-efficacy stemmed from worries about following the insulin regimen in novel situations such as during festive meals, which are a common occurrence in Malaysian culture.

"“…there is a lack of self-efficacy…Now self-efficacy is mainly can you handle in a s-situation, in a situation that you are in. And is not just what you can do, can you handle it? Even if you know how to give yourself injection, if you have to go for some s-social function. What do you do? What do you do?”"

"Diabetes nurse educator, private practice"

Being elderly, relying on others for care, suffering from visual impairment and having irregular mealtimes also caused patients to hesitate over starting insulin.

"“For my patient, like elderly, we have resistant to start insulin because cannot read the pen- too small and then blur.... So, because cannot see, cannot read…they got the eye problem”"

"Family medicine specialist, public health centre"

The healthcare professionals felt that some patients lacked knowledge and were reluctant to start insulin, especially those who had a short history of diabetes. Some were not aware of the natural progression of diabetes and the need for insulin eventually.

"“In fact probably they’re already diabetes for many years but just diagnosed for two years. So they thought, you know, it’s…it’s just too early. It’s just too early for them to actually go for insulin.”"

"Family medicine specialist, public health centre"

#### Negative influence from family members

Another barrier noticed by the health care professional is some patients with diabetes were facing negative influence and poor support from family members especially from their spouse to initiate insulin.

"“(Patient) agreed to have insulin…Next day he came back and said “My wife doesn't want me to…start insulin”…My personal feeling is that he’s completely under her thumbs, and she has decided “My husband doesn’t need insulin”."

"Endocrinologist, private hospital"

### Healthcare professional barriers

Healthcare professional barriers to initiating insulin therapy comprised psychological barriers such as negative attitudes towards insulin therapy, lack of motivation and confidence. Unfamiliarity with starting insulin therapy was also highlighted.

#### Negative attitudes towards insulin

Some healthcare professionals felt that it was unlikely for patients to change their negative attitudes towards insulin and to modify their lifestyle to suit the insulin regimen. These healthcare professionals were unwilling to take time to teach patients about insulin therapy, and viewed insulin as a hassle.

"“I also discuss (insulin) with, um…the FMS (Family medicine specialists) or in Terengganu and the physicians…and a matter of factly it’s…it’s as if they just accept the fact (that patients won’t start insulin). “It’s difficult here! The patient doesn’t want to do, what can we do…Patient don’t want insulin, so what can we do?”"

"Government policy maker"

#### Lack of motivation and confidence

Some doctors were not motivated to start patients on insulin themselves as they could refer patients to an endocrinologist or a diabetic nurse. Furthermore, some doctors still subscribed to the old school of thought that insulin could only be initiated in a hospital setting and not in clinics.

"“I don’t push… I don’t push, because I let the specialists handle it. Yeah, I refer them to the specialists…”"

"General practitioner, private practice"

Besides motivation, some doctors lacked confidence in starting a patient on insulin. Reasons included feeling uncomfortable with needles and unfamiliarity with the various insulin regimens and devices. Some healthcare professionals blamed the patients for their reluctance to accept insulin. Even those who were successful at initiating insulin viewed the counselling process as a battle to be won and one requiring considerable mental preparation.

"“…we ourselves have got such a mental block. I mean, as doctors it’s very easy to preach, but when it comes to needles I think we doctors also freak out. So when we had to inject it was like, ‘Oh dear…must I do it?’”"

"General practitioner, private practice"

#### Training-related barriers

Training-related barriers include: organizational policies that do not support staff who want further training and the quality of the training programme.

"“…even though they (sponsors) write there black and white for the (diabetes) educator from the clinic to go (for training)… we are not at liberty to improve ourselves”"

"Diabetes nurse educator, public university hospital"

"“…the training in the Ministry (of Health) is very much didactic, not so much practical.”"

"Government diabetes policy maker"

#### Conflicting advice from the healthcare professionals

Conflicting information given by healthcare professionals, peers and media tended to delay patients’ decision in starting insulin.

"“…the GP told him…“No, why you so silly start on injection for? I give you medicine. Forget it, throw it all away.” So he went back to oral medicine....and he came back 6 months later with renal failure.”"

"General practitioner, private practice"

Doctors in the private sector felt that the decision to start insulin or not was out of their control as patients could *“shop with another doctor who will tell them that they don’t need (insulin)”* (Diabetes nurse educator, private practice).

### System barriers

System barriers to insulin initiation could be divided into four main areas: lack of continuity of care, manpower, resources and language barriers.

#### Lack of continuity of care

The lack of continuity of care in primary care made insulin initiation and management challenging. Therefore, patients were often unable to maintain the follow-up which is crucial to address individual patient’s concerns about insulin. The lack of continuity of care is particularly problematic in the public sector due to high turnover of doctors. Patients are often not being given a choice on who they would like to consult as they are unable to book to see the same practitioner at each visit.

"“So, I’ve learned that it’s important to…to…to know your patient well but the only problem with MOH (Ministry Of Health) is that you can’t see the same doctor…… so this fact about not having the same doctor, patients don’t like it. They don’t like it.”"

"Endocrinologist, public hospital"

#### Lack of manpower

The lack of manpower was apparent especially in the government hospitals and clinics. Despite recognising the important role of a nurse educator in insulin counselling, only a small number of diabetes nurse educators and dieticians were trained in the government sector and, when present, they had to handle heavy patient loads. Although privately-sponsored diabetes nurse educators were available to help educate patients on starting insulin in private clinics, there were very few of them.

#### Lack of resources

While insulin is subsidized in the public clinics and hospitals, there is no financial assistance for glucometers and test strips. This hampers insulin initiation.

"“The other thing is that I think, uhh…most of our patient do not have home blood sugar monitoring. This is actually very difficult in starting insulin. To actually titrate insulin, especially for BIDS (bedtime insulin daytime sulphonylurea) regime, it’s very difficult.”"

"Family medicine specialist, public health centre"

Education materials about insulin were not easily available and most insulin-prescribing doctors preferred to sketch out information on blank paper. There was also a lack of dedicated diabetes education rooms and facilities. Counselling patients about insulin initiation was seen as time consuming especially in government clinics with heavy patient load.

"“…the workload…500, 600 patient a day and per doctor we are seeing umm, 70 to 100. Not a good day, one MC (medical leave), one taking leave, 100 a day. So I was you know, practicing there, I have to be a regular MO (Medical Officer), so I can find it is difficult to counsel patient in this kind of situation. Time is definitely you know really un-under constraint”"

"Family medicine specialist, public health centre"

#### Language barriers

Language issues made it difficult for healthcare professionals to communicate with patients. Some patients from rural and agricultural estates can only speak their native language. This poses a big communication barrier if the healthcare professional and the patient are from different ethnic and linguistic backgrounds.

"“…the big, important issue is language barrier…we actually do not have enough…uh…Indian staff.”"

"Family medicine specialist, public health centre"

## Discussion

This study highlights the wide range of barriers to insulin initiation in Malaysia and provides an overview as to why the use of insulin remains low. What is remarkable is the similarity of the barriers encountered in a multicultural, Asian country to barriers reported in studies conducted in the West. A Pubmed search of qualitative studies which focus on barriers to insulin initiation identified eight studies from North American [25,26], UK [27-30], European [31] and South African [32] settings. Thematic consistency is apparent between these studies and our study, suggesting that these barriers are widely held ideas and that the results of this study are generalisable.

Studies on psychological insulin resistance amongst multi-ethnic populations have found that ethnicity is an important determining factor. Studies in the west have found that Hispanic and ethnic minorities are less willing to start insulin therapy [19,33]. Reasons for this resistance include perceived lack of access to care and language barriers between healthcare provider and patient [34,35]. Malaysian society consists of three main racial groups, each with distinct cultural practices and close-knit community structures. The healthcare professionals cited patients’ misconceptions of insulin as a major barrier. Our study identified three misperceptions that arise out of this multicultural setting: religious barriers, use of complementary medicines and lethal connotations about insulin.

The majority of Malaysia’s population are Muslim, in which the origin of food and products must comply to strict religious standards in order to be considered lawful (‘halal’). Healthcare professionals need to reassure Muslim patients that modern, synthetic insulin is not derived from a porcine source [36], which is strictly forbidden except under emergency situations [37]. Another concern for Muslim patients is the use of insulin during Ramadan, where the Muslims would be on a full-day fast from food and drink [38]. Healthcare professionals (including non-Muslims) must be able to advise Muslim patients on appropriate insulin regimes during the fasting month of Ramadan [39].

Patients’ preference to try out complementary therapies before insulin usage is often overlooked by the healthcare professionals in Malaysia. In a local study, the use of complementary therapies was prevalent among people with type 2 diabetes mellitus [40]. Half of Malaysian patients with chronic diseases do not report their use of complementary therapy to their doctors or pharmacists [40]. This is of concern as the use of traditional herbs has been identified elsewhere as a barrier to insulin therapy whereby patients were perceived to have more faith in herbs than in insulin [32]. Increasing healthcare professional awareness on complementary and traditional therapies will help to reduce healthcare professionals’ anxiety in advising patients on the use of such therapies [41]. Healthcare professionals need to play a more active role in asking their patients about their use of complementary therapies when initiating insulin [42].

Patients often associate insulin usage with co-morbidities. Although it has been reported elsewhere that patients associate insulin with disease severity [25,43], this misconception appeared to be more serious among the Malaysian patients who consider insulin to be lethal. Healthcare professionals should, therefore, address this misconception by counselling patients about the natural progression of diabetes at early stage of the illness. It should be emphasised to patients that early initiation of insulin helps to reduce morbidity and mortality. The myth about the association between insulin and advanced disease and deaths should be dispelled by providing accurate and timely information to the patients.

In this study, most system barriers are similar to those found elsewhere, including short consultation times, rapid staff turnover and lack of continuity of care [32]. However, further matrix analysis of the data identified two issues which were only identified in healthcare professionals from the public healthcare system in Malaysia. Firstly, the lack of continuity of care is particularly problematic in the public healthcare setting due to fast turnover of doctors and patients not being given a choice on who they would like to consult. Continuity of family physician care in patients with diabetes is associated with better quality of life [44], and lower mortality and hospitalization in elderly patients [45]. According to Prochaska’s transtheoretical model [46], insulin initiation requires patients to move from stages of precontemplation, contemplation and finally to action, with patients often cycling back and forth between these stages [47]. Continuity of care would play an important role as healthcare professionals assess the stage of patient’s readiness to initiate insulin and customize a follow-up plan to help patients initiate and optimize the use of insulin [48].

The language barrier was especially pressing in rural and semi-rural locations of the public healthcare system. Patients with limited language proficiency have problems with healthcare access, comprehension, adherence and receive lower quality of care overall [49]. As a self-administered injection, insulin requires an understanding of injection techniques and self-titration. Thus, difficulty in communication during patient education still poses a substantial barrier to insulin initiation in Malaysia. Strategies to overcome language barriers in practice include employing a diverse healthcare workforce and using translation services when necessary [50]. Preparing healthcare professionals to serve in diverse communities can be done by offering medical language courses in medical schools to help familiarise students with medical terminologies they will encounter in different communities [51].

Both public and private healthcare professionals stated that the lack of resources was an important barrier to insulin initiation. Diabetes nurse educators are an important, but lacking resource for insulin initiation, with less than 600 diabetes nurse educators in the country serving a diabetes population of approximately 1.6 million [52]. The cost and lack of availability of self monitoring of blood glucose (SMBG) contribute to patients’ reluctance to start insulin. Although the cost of insulin is subsidized in Malaysia, glucometers and test-strips are not. There is evidence to suggest that the frequency of SMBG is inversely related to out-of-pocket expenses [53,54] and countries with the highest relative strip-cost have the lowest use of self-monitoring [55]. Thus, one place to start is to look into providing patients with financial assistance to acquire glucometers and test-strips for SMBG as they are essential for monitoring the response to and side effects of insulin therapy.

Patients perceive that their diabetes is advanced once they are advised to start insulin therapy [19]. This perception may stem from the healthcare professionals’ belief that insulin could only be started once the patients reach maximum numbers and doses of oral glucose-lowering drugs. Previous Malaysian CPGs recommended that insulin should only be considered in patients with poor glycaemic control after lifestyle modifications and maximum oral glucose-lowering therapy [56]. In the latest CPG released in 2009, the recommendation has been changed and healthcare professionals are now advised to start insulin early, especially for patients who have poor glycaemic control at diagnosis. More research is needed on the prevalence of the ‘legacy effect’ of past guidelines and changes made from previous guidelines should be highlighted during the training and dissemination of new guidelines [57].

The strength of this study lies in the fact that the sample encompassed all healthcare sectors and stakeholders who were involved in insulin initiation. We were thus able to gain an in-depth understanding of the barriers to insulin initiation from a wide range of perspectives. Analysis of barriers according to participant ethnicity did not reveal significant differences in terms of themes mentioned as healthcare professionals treat patients from various ethnicities and encounter a range of barriers in patients. However, participant responses highlighted the nature of culture-specific barriers as the examples provided were often specific to one culture, such as the names of traditional herbs.

There are a few limitations in this study. Only participants from three states (Kuala Lumpur, Selangor and Seremban) in Malaysia were included in this study. The culture of patients in other states, in particular the East coast of the peninsula and East Malaysia, might be different and hence the patients might face different barriers when starting insulin. This limits transferability. Future studies should include participants from other states of Malaysia. As sample size was determined by thematic saturation, the sample population was too small to be analysed according to healthcare professions. Lastly, only healthcare professionals’ perspectives were included for this study. However, this study forms part of a larger study and we are embarking on a study exploring patients’ views and perceived barriers to starting insulin. More research is necessary to explore the patients’ perspectives of insulin therapy. This will help substantiate the findings from this study and identify the needs of patients when starting insulin.

## Conclusions

Tackling the issue of insulin initiation should not happen only at the point of decision during clinical consultations. A more comprehensive healthcare education programme should be designed and implemented. Patients should be informed early on about the natural progression of diabetes and the need for insulin therapy 10–15 years after the diagnosis. At the macro level, understanding the barriers to insulin initiation helps government policy makers develop effective public educational programmes; design and implement training curriculum of healthcare professionals; and plan the resources necessary to manage this disease. At the micro level, the awareness of the barriers to insulin initiation helps the healthcare professionals to explore and address patients concerns and help them to make an informed decision about insulin initiation.

## Misc

Yew Kong Lee, Ping Yein Lee and Chirk Jenn Ng are contributed equally to this work.

## Competing interests

The author(s) declare that they have no competing interests.

## Authors’ contributions

YKL, PYL and CJN were involved in developing the interview topic guides used for data collection, facilitating the data collection interviews and focus groups, and analysing the data for reporting. All authors read and approved the final transcript.

## Pre-publication history

The pre-publication history for this paper can be accessed here:

http://www.biomedcentral.com/1471-2296/13/28/prepub
